# Viruses and Endocrine Diseases

**DOI:** 10.3390/microorganisms11020361

**Published:** 2023-01-31

**Authors:** Magloire Pandoua Nekoua, Cyril Debuysschere, Inès Vergez, Corentin Morvan, Chaldam Jespere Mbani, Famara Sane, Enagnon Kazali Alidjinou, Didier Hober

**Affiliations:** Laboratoire de Virologie ULR3610, Université de Lille, CHU Lille, 59000 Lille, France

**Keywords:** virus, infection, endocrine system, endocrine diseases

## Abstract

Viral infections have been frequently associated with physiological and pathological changes in the endocrine system for many years. The numerous early and late endocrine complications reported during the current pandemic of coronavirus disease 2019 (COVID-19) reinforce the relevance of improving our understanding of the impact of viral infections on the endocrine system. Several viruses have been shown to infect endocrine cells and induce endocrine system disturbances through the direct damage of these cells or through indirect mechanisms, especially the activation of the host antiviral immune response, which may lead to the development of local or systemic inflammation or organ-specific autoimmunity. In addition, endocrine disorders may also affect susceptibility to viral infections since endocrine hormones have immunoregulatory functions. This review provides a brief overview of the impact of viral infections on the human endocrine system in order to provide new avenues for the control of endocrine diseases.

## 1. Introduction

Viruses are small obligatory intracellular parasites with an RNA or DNA genome, and they infect a variety of eukaryotic or prokaryotic cells. The viruses that infect humans are extremely diverse, and they are responsible for acute or chronic diseases that can occasionally reach pandemic proportions, such as the current pandemic of coronavirus disease 2019 (COVID-19). Viruses enter host cells after recognition and binding to host-cell-specific receptors, and they modulate cellular functions in order to replicate and produce progeny virions capable of infecting other cells and spreading in the host. Structural or functional cellular alterations due to viral replication and the activation of the host antiviral immune response may lead to the development of local inflammation and/or the destruction of infected cells/tissues or systemic inflammation that results in the dysfunction of multiple organs, including those of the endocrine system [1].

The endocrine system is a complex interconnected system of hormone-producing cells/organs that play roles in maintaining homeostasis and in modulating the immune response to infections. Several epidemiological and clinical studies have reported multiple endocrine and metabolic abnormalities following virus infections, such as human immunodeficiency virus type-1 (HIV-1), coxsackieviruses B (CVB), and severe acute respiratory syndrome coronaviruses (SARS-CoV) [2,3,4,5,6]. In addition, it has also been suggested that endocrinopathies, such as adrenal insufficiency, type 1 and 2 diabetes, and Cushing’s syndrome, increase the risk of SARS-CoV-2 infection and the critical clinical progression of COVID-19 [7].

Several viruses have been shown to infect endocrine cells in vitro, ex vivo, and in vivo [1,5,8,9,10] and to induce endocrine system disturbances through the direct damage of these cells or through the cytokine-mediated activation of the hypothalamic–pituitary–adrenal (HPA) axis in particular [11].

This review provides a brief overview of the impact of viral infections on the human endocrine system in order to provide new avenues for the control of endocrine diseases.

## 2. Coronaviruses and the Endocrine System

Human coronaviruses are enveloped, positive-sense, single-stranded RNA viruses belonging to the *Coronaviridae* family. SARS-CoV-2, which caused the COVID-19 pandemic, shares an 80% identity with SARS-CoV and a 50% identity with Middle East respiratory syndrome coronavirus (MERS-CoV) [12]. SARS-CoV and SARS-CoV-2 enter target cells through the binding of the viral spike (S) protein to the cellular receptor angiotensin-converting enzyme 2 (ACE2) and after S protein priming by the host cell transmembrane serine protease 2 (TMPRSS2) [13]. In humans, ACE2 and TMPRSS2 mRNAs are expressed in several endocrine tissues, including the hypothalamus; the pituitary, thyroid, and adrenal glands; the ovaries; the testes; and the pancreatic islets [8].

The HPA axis is a major neuroendocrine system involved in the maintenance of resting and stress-related homeostasis through the release of corticotropin-releasing hormone (CRH) from the hypothalamus; this stimulates the release of adrenocorticotropic hormone (ACTH) from the anterior pituitary gland, which, in turn, stimulates the secretion of cortisol (one of the major glucocorticoids) from the adrenal glands. The main HPA axis dysfunction in SARS-CoV survivors 3 months after recovery is central hypocortisolism (39% of cases with low ACTH levels), which resolves within one year in 62% of patients [14]. Alterations in adenohypophyseal endocrine cells have been reported in patients with SARS-CoV infection and have been found to be consistent with the increased serum levels of prolactin, follicle-stimulating hormone (FSH), and luteinizing hormone (LH) and the decreased serum levels of growth hormone (GH), thyroid-stimulating hormone (TSH), and adrenocorticotropic hormone (ACTH) [15]. Similar disturbances have been observed in patients with SARS-CoV-2 infection with respect to LH, prolactin, GH, and TSH [16,17]. Hyponatremia has been reported among patients with COVID-19 and may be due to the syndrome of inappropriate antidiuretic hormone secretion [18]. This electrolyte disorder is thought to be associated with excess serum levels of interleukin-6 (IL-6) during COVID-19, which stimulate the HPA axis to induce the non-osmotic release of vasopressin [18,19]. Several case reports have suggested that COVID-19 could be a risk factor for pituitary infarction since SARS-CoV-2 can induce coagulopathy, platelet dysfunction, and thrombocytopenia [6,20]. Furthermore, hypopituitarism, Cushing disease, and adrenal insufficiency may represent risk factors for severe COVID-19 in infected patients [21]. Wheatland suggests that adrenal insufficiency during SARS-CoV infection may be the result of a viral strategy to evade the immune system, such as the inhibition of the host’s corticosteroid stress response. Indeed, the molecular mimicry between the amino acid sequences of SARS-CoV and host ACTH may induce the host immune system to produce antiviral antibodies that are similar to anti-ACTH autoantibodies and that may interfere with the ability of ACTH to stimulate corticosteroid secretion from the adrenal glands [22]. It is not excluded that SARS-CoV-2 uses the same molecular mimicry strategy.

The prevalence of hypothyroidism has been found to be low in patients infected with SARS-CoV, as well as in those infected with SARS-CoV-2 (5–6%) [14,23]. Thyroid lesions, including alterations in follicular and parafollicular cells, have been reported in patients infected with SARS-CoV [24], and this is consistent with the decreased thyroxine and triiodothyronine serum levels frequently reported in these patients [14]. In contrast, no significant thyroid follicle lesions have been found in patients infected with SARS-CoV-2 [25,26]. However, thyrotoxicosis (15–20% of cases) and low serum TSH and 3,5,3′-triiodothyronine levels (compared with those of a healthy control group) have been reported in patients with COVID-19, and they have been found to be significantly associated with increased IL-6 serum levels in these patients and with the severity of the disease [16,23,27,28]. A few cases of subacute thyroiditis [29,30,31,32] and Grave’s disease [33,34] have also been reported in patients with COVID-19, but no data on subacute thyroiditis associated with the SARS-CoV outbreak have been found.

Clinical reports have suggested that preexisting diabetes mellitus is a common comorbidity observed in 10–22% of patients with COVID-19 [35,36,37] and that it is associated with COVID-19 severity and increased mortality [38,39]. ACE2 and TMPRSS2 mRNAs or proteins are highly expressed in human pancreatic islets, as well as in ductal and acinar cells [8,40,41,42,43]. A recent U.K. multicenter study and a meta-analysis have provided evidence that new-onset type 1 diabetes (T1D) in children increased during the COVID-19 pandemic [44] and that severe COVID-19 is associated with increased blood glucose levels [45]. COVID-19 is also associated with ketoacidosis (with 77% of cases occurring more frequently in patients with pre-existing type 2 diabetes (T2D)) [46], pancreatitis, and increased amylase or lipase levels, suggesting that SARS-CoV-2 infection may affect the exocrine pancreas [47]. SARS-CoV and SARS-CoV-2 have been detected in the pancreas samples (exocrine and endocrine cells) of patients who died from SARS and COVID-19 via immunohistochemistry, in situ hybridization, and immunofluorescence [9,48,49]. Interestingly, it has recently been shown that SARS-CoV-2 can replicate in human pancreatic islets ex vivo, resulting in the impairment of β-cell function, including impaired glucose-stimulated insulin secretion and reduced numbers of insulin-secretory granules in β-cells [9,49].

ACE2 receptor expression is found in testicular germ cells, Leydig cells, and Sertoli cells [50], as well as in ovarian tissues, the uterus, the placenta, the vagina, and the breasts [51,52]. Testes are susceptible to damage by SARS-CoV-2 infection. Indeed, SARS-CoV-2 has been detected in testis autopsies [53] and in the semen obtained from both patients with COVID-19 and those who are recovering [54]. In addition, men with COVID-19 have been found to have decreased serum sex hormone levels, including total testosterone, compared with controls, suggesting defective Leydig cell function [55,56,57,58]. Low testosterone levels in hospitalized patients infected with SARS-CoV-2 have been found to be inversely associated with markers of inflammation, including IL-6 and C-reactive protein, and they have been found to be associated with increased disease severity [56], suggesting the involvement of immune-mediated mechanisms. In a prospective Chinese study, a significant decrease in serum anti-Müllerian hormone and increases in serum total testosterone and prolactin levels were reported in women with COVID-19 compared to an age-matched healthy control group [59]. SARS-CoV-2 infection has been found to be associated with changes in menstrual volume (25%) and cycle prolongation (19%) in women diagnosed with COVID-19, which may be consequences of transient changes in sex hormones during the disease [60]. Menstrual disturbances, especially irregular menstruation and abnormally heavy periods and postmenopausal bleeding, have also been reported in 36% of women with long COVID [61].

It is interesting to note that some drugs used in the management of patients infected with SARS-CoV-2 may affect the endocrine system. Glucocorticosteroids and low-molecular-weight heparin (LMWH) have been used extensively around the world in the management of severe and critical COVID-19 [62]. Glucocorticoid treatment is known to induce hyperglycemia, insulin resistance, and dyslipidemia [63]. Long-term corticosteroid therapy may also inhibit LH and FSH secretion and lead to secondary osteoporosis and the suppression of adrenal hormones, which may result in adrenal insufficiency after the cessation of treatment [4,63] and impact the severity of COVID-19 [7]. Several studies have shown that pharmacological doses of glucocorticoids can inhibit the secretion of TSH and the peripheral conversion of thyroxine (T4) to triiodothyronine (T3) in humans, which return to normal after the cessation of treatment [6,64]. It has been suggested that glucocorticoids can suppress the release of TSH from anterior pituitary thyrotrophs through the inhibition of TSH-releasing factor in the hypothalamus or through the protein kinase C-dependent phosphorylation of the protein annexin 1 [21,64,65]. LMWH therapy, used to prevent hypercoagulability associated with severe SARS-CoV-2 infections, may interfere with the measurement of serum-free thyroid hormones, which may show a false elevation [64]. Therefore, it seems important to follow up patients with endocrine alterations that occurred during the course of SARS-CoV-2 infection.

## 3. Human Immunodeficiency Viruses and the Endocrine System

Human immunodeficiency viruses (HIVs) are RNA retroviruses that belong to the *Lentivirus* genus of the *Retroviridae* family. They are the causative agents of Acquired Immune Deficiency Syndrome (AIDS), which remains a public health problem, as 38.4 million people worldwide were living with HIV in 2021 [66].

Patients infected with HIV often develop abnormal body fat distribution associated with insulin resistance, hyperlipidemia, increased free fatty acids [67,68], systemic inflammation [69], and alterations in growth hormone secretion [70,71,72]. Case reports have suggested that HIV infection is associated with hypopituitarism [73,74], diabetes insipidus [74], adrenal insufficiency [74,75], and hypothyroidism [76]. A prospective study of endocrine function in patients infected with HIV reported an abnormal cortisol response to ACTH in 7% of patients infected with HIV, and it reported testosterone deficiency and euthyroid sick syndrome in 28% of male patients with AIDS and in 16% of patients with AIDS, respectively [77]. Hypogonadism has been reported in men infected with HIV (16%) and in women infected with HIV (25%) since the beginning of the HIV epidemic [3,78,79,80], and this may be caused by multifactorial events, including the presence of opportunistic infections affecting the pituitary gland or hypothalamus, chronic systemic illnesses, weight loss, undernutrition, and direct cytokine effects on the gonads [3,79,80]. The pituitary gland appears to be affected at various stages of HIV infection. Indeed, it has been shown that mean basal serum GH, prolactin, and testosterone concentrations are similar in subjects positive for HIV (with or without AIDS) as compared to controls, whereas the basal serum concentrations of TSH, LH, ACTH, and cortisol are increased in subjects with AIDS [81]. In addition, the poststimulation maximum levels of GH, prolactin, TSH, and ACTH have also been found to be increased in this group, which suggests an increased activity of the pituitary gland; however, the mechanism remains unknown [81].

Insulin resistance and T2D have been described in patients infected with HIV, but the link between the HIV infection itself and an increased risk of T2D is controversial [82,83,84]. However, it has been shown that the systemic inflammation present in patients infected with HIV is associated with the risk of developing T2D [85]. In addition, alterations in adipokine levels and CD4+ and CD8+ T-cell function and increased microbial translocation are related to insulin resistance, lipodystrophy, dyslipidemia, and glucose metabolism alterations in patients infected with HIV [86,87,88,89]. The markers of systemic inflammation decrease quickly with antiretroviral therapies (ARTs) [90], but this remains insufficient to reduce the risk or prevalence (2–14%) of diabetes mellitus in patients infected with HIV on therapy [82,83,85,91,92].

Endocrine function appears to be relatively preserved in most cases of patients infected with HIV on ARTs, but these drugs can also have adverse effects on the endocrine system, including insulin resistance, diabetes mellitus, dyslipidemia, hypogonadism, and osteoporosis [2,3]. One of the most widely described effects of protease inhibitors and non-nucleoside reverse transcriptase inhibitors is the development of insulin resistance and diabetes mellitus through the inhibition of glucose transporter type 4 and insulin secretion, as well as through their effects on subcutaneous fat and mitochondrial toxicity [3]. However, the most recent protease inhibitors (darunavir and atazanavir) have a much less diabetogenic effect [2]. Other alterations associated with ARTs have also been described, such as secondary hypogonadism; dyslipidemia; altered fat distribution; and alterations in TSH, prolactin, FSH, and T3/T4 levels [2,3,74]. Increases in prolactin and altered bone mineralization have been described with some protease inhibitor treatments [3]. Some ARTs may affect cortisol metabolism or inhibit cytochrome P450 enzyme 3A4 activity, which may precipitate adrenal insufficiency and Cushing syndrome [2,3]. Some other drugs used to treat HIV-associated complications can also cause endocrine disruption. Ketaconazole, an antifungal drug, increases steroid clearance and can therefore induce hypogonadism in men or menstrual cycle disturbances in women [2,3]. Megestrol acetate, sometimes used in HIV-associated cachexia, can cause adrenal insufficiency [2,3]. These considerations must be balanced against the potential pathogenic effect of HIV infection on the endocrine system.

## 4. Hepatitis Viruses and the Endocrine System

Hepatitis B virus (HBV) and hepatitis C virus (HCV) are a DNA virus of the *Hepadnaviridae* family and an RNA virus of the *Flaviviridae* family, respectively. Chronic HBV and HCV infections are common predisposing factors leading to liver fibrosis, cirrhosis, and hepatocellular carcinoma, but they have also been associated with extrahepatic manifestations, including endocrine disorders, such as autoimmune thyroiditis (characterized by the lymphocytic infiltration of the thyroid), T2D, and erectile dysfunction [93].

Retrospective and prospective studies and meta-analyses strongly support the association between chronic HCV infection and a high prevalence of hypothyroidism, anti-thyroperoxidase antibodies (TPOAb), anti-thyroglobulin antibodies (TgAb), and papillary thyroid cancer [94,95,96,97,98]. For example, patients with chronic HCV infection are more likely to have hypothyroidism (13%), TPOAb (21%), and TgAb (17%) than controls or patients with chronic HBV [99]. High serum TSH levels and low serum FT3 and FT4 levels have also been found in these patients compared to controls or patients with chronic HBV [99].

It has been shown that HCV can infect a human thyroid cell line in vitro [100], and increased expressions of IFN-γ and CXCL10 have been reported in the hepatocytes and lymphocytes of patients infected with HCV [101,102]. Therefore, Antonelli’s team speculated that thyroid infection by HCV could upregulate CXCL10 gene expression and secretion in thyrocytes, leading to the infiltration of the thyroid by Th1 lymphocytes that secrete IFN-γ and TNF-α [103,104]. This HCV-induced inflammatory process may lead to the destruction of thyroid follicular cells and to the appearance of autoimmune thyroiditis or thyroid cancer [103,104].

Two meta-analyses of prospective and retrospective studies have suggested that HCV infection is associated with an increased risk of T2D in patients with chronic HCV, especially in patients infected with HCV with cirrhosis [105,106]. It has been estimated that between 13 and 33% of patients with chronic HCV have diabetes in several regions of the world [107]. The data on the association between HBV infection and an increased risk of T2D are controversial [105,106,108]. However, it was reported that the prevalence of patients infected with HCV is higher (5.9%) than that of patients infected with HBV (1.6%) in an Italian cohort of patients with diabetes [109]. In addition, the adjusted hazard ratio for diabetes development was found to be higher in patients co-infected with HCV/HBV (1.90) than in patients infected with HCV and in patients infected with HBV in Korean population-based cohort data (1.68 and 1.41, respectively) [110]. The underlying mechanisms of the involvement of HCV infection in the development of diabetes have been suggested. Indeed, HCV replication can impair glucose uptake in human hepatic cell line and primary hepatocytes by downregulating the cell surface expression of GLUT2 [111], which can promote hyperinsulinemia and insulin resistance. Moreover, an excessive TNF-α response and high oxidative stress markers, such as the serum and liver thioredoxin levels reported in patients infected with HCV, have been associated with insulin resistance and the development of T2D [107,112]. At the same time, diabetes and insulin resistance have been shown to be independent factors associated with the progression of liver fibrosis and cirrhosis, as well as with an increased risk of developing hepatocellular carcinoma in individuals infected with HCV [113]. Maternal chronic HBV or HCV infection has been shown to have long-term effects on endocrine morbidity in offspring; specifically, higher rates of hypoglycemia have been found in the offspring of mothers infected with HCV (1.1%) than in the offspring of mothers infected with HBV (0.2%) and in the offspring of non-infected mothers (0.1%) [114].

Sexual impotence and alterations in spermatogenesis also appear to be consequences of chronic HCV infection. About 30% of male patients with chronic HCV infection, especially those with liver cirrhosis, have erectile dysfunction (ED) [115]. Significant increases in homocysteine and estrogen levels and a reduction in insulin-like growth factor 1 levels have been observed in patients with ED associated with chronic HCV infection, and a strong association between the severity of ED and chronic HCV has been demonstrated [116]. Patients with chronic HCV infection have low serum levels of inhibin B and total testosterone, as well as abnormal sperm parameters (a decreased sperm volume, sperm count, and sperm motility), compared to controls, suggesting a possible negative influence of the virus on spermatogenesis [117,118].

The treatment of HCV infection has long been based on the use of interferon-α (IFN-α). This drug is mainly associated with several adverse effects on the thyroid gland [119]. Thyroid dysfunction appears to be more common in patients infected with HCV treated with IFN-α than in patients infected with HBV on the same therapy [119]. Many thyroid disorders (hypo- and hyper-thyroidism) have been described, sometimes with increased levels of anti-thyroid antibodies in patients infected with HCV treated with IFN-α [63,119]. Anti-21 hydroxylase antibodies have been found in some patients infected with HCV receiving IFN-α therapy, but no clinical adrenal insufficiency has been reported [119]. The development of T1D and hypopituitarism seem rare and doubtful in patients infected with HCV receiving IFN-α therapy [119,120]. However, current protocols tend to use direct-acting antivirals in HCV-related diseases, which have been shown not to affect thyroid function or to trigger autoimmunity [121,122].

## 5. Orthohantaviruses and the Endocrine System

Orthohantaviruses, previously known as Hantaviruses, are zoonotic, enveloped, single-stranded, negative-sense RNA viruses belonging to the *Orthohantavirus* genus and the *Hantaviridae* family [123]. Rodent-borne orthohantaviruses have a diverse worldwide distribution and can cause severe diseases in humans, such as hantavirus pulmonary syndrome and hemorrhagic fever with renal syndrome (HFRS), which can reach mortality rates of 12% and 60% during certain outbreaks, respectively [124]. HFRS is caused mainly by *Murinae*-borne orthohantaviruses, such as Hantaan virus, Seoul virus, and Dobrava virus, as well as by *Arvicolinae*-borne orthohantaviruses, such as Puumala virus (PUUV) [125].

Orthohantaviruses are able to infect endothelial cells, macrophages, and renal glomerular and tubular cells in vitro [126,127], and they have been detected in the pituitary stromal and vascular endothelial cells, renal tubuli, and spleens of post-mortem samples from patients with nephropathia epidemica due to PUUV infection [128]. Several case reports have described hypopituitarism during or months after orthohantavirus infection, with necrotic and hemorrhagic damage of the pituitary gland being confirmed via radiographic imaging in 58 to 72% of patients with HFRS [128,129,130,131]. Hypotension and/or vasospasms during the acute phase of HFRS, thrombocytopenia, thrombopathy, and other known causes of coagulation disorders during orthohantavirus infection have been suggested to be the main pathophysiological mechanisms leading to the pituitary damage [132]. Some case reports have also suggested that hormonal defects and abnormalities of the gonadal and/or thyroid axis reported during or after PUUV infection [133,134,135,136] may develop due to an autoimmune mechanism [135].

## 6. Human Parvovirus B19 and the Endocrine System

The primate erythroparvovirus 1, previously known as human parvovirus B19 (PVB19) or erythrovirus B19, is a small, non-enveloped DNA virus that belongs to the *Erythroparvovirus* genus of the *Parvoviridae* family [123]. It is an ubiquitous virus that is transmitted mainly through the respiratory tract and blood transfusion, and vertical transmission can also occur. Acute PVB19 infection has been shown to be a cause of erythema infectiosum in children, erythroblastopenia crisis, and hydrops fetalis [137]. PVB19 is also suspected to be an environmental factor involved in the pathogenesis of autoimmune thyroid diseases (AITD), including Graves’ disease (GD) and Hashimoto’s thyroiditis (HT).

In a previous study, PVB19 viremia was detected via PCR in 12% of children with HT versus 3% controls, and it was suggested that acute PVB19 infection could be associated with the appearance of HT [138]. Moreover, PVB19 DNA or the capsid protein have been detected in human thyroid tissues and, more frequently, in papillary thyroid carcinoma and HT tissues via nested PCR, in situ hybridization, and immunohistochemistry [139,140,141]. In contrast, PVB19 has been detected specifically in the thyroid follicle cells of thyroidectomy specimens, but it has not been more frequently detected in the thyroids of patients with GD in comparison to non-autoimmune multinodular thyroids [142,143]. In addition, the upregulation of the positive regulatory domain zinc finger protein 1 (PRDM1) has been demonstrated in primary thyroid epithelial cells after PVB19 NS1 transfection [144]. The persistence of PVB19 DNA in the thyroid of patients with AITD has been reported [145,146], and it has been suggested that it may initiate the intrathyroidal inflammatory process [145]. No direct evidence formally demonstrating the role of PVB19 infection in the pathogenesis of AITD has been obtained to date, but some pathophysiological hypotheses have been suggested, including cell apoptosis and increased inflammatory-related gene expression induced by the virus genome or protein [147,148,149]. Further studies are needed to determine the role of PVB19 in AITD.

## 7. Enteroviruses and the Endocrine System

Enteroviruses (EVs) (viruses of the genus *Enterovirus*) are small, non-enveloped, positive-sense, single-stranded RNA genome viruses belonging to the *Picornaviridae* family. T1D, which results from an autoimmune attack and a loss of insulin-producing β-cells of the pancreas, is the major widely documented endocrine disease associated with EV. The markers of EV infection (the VP1 capsid protein or viral RNA) are more frequently detected in the serum, monocytes, intestinal mucosa, and pancreas of patients with type 1 diabetes than in controls in many parts of the world [150,151,152,153,154,155]. Two meta-analyses that included 4448 and 5921 participants statistically confirmed the association between the presence of these enteroviral infection markers and the development of anti-islet autoantibodies and the development of T1D (odds ratios = 9.8 and 7.8, respectively) [156,157]. Epidemiological and experimental studies have suggested that coxsackievirus B (CVB) is among the enterovirus species most likely to be involved in the pathogenesis of T1D [5,158]. CVB can initiate autoimmunity against pancreatic β cells through several mutually non-exclusive mechanisms, including (i) molecular mimicry between conserved enteroviral proteins and pancreatic β-cell proteins, (ii) the bystander activation of pre-existing autoreactive T cells, (iii) alterations in the central tolerance to β-cell antigens resulting from thymus infection, (iv) the production of non-neutralizing antibodies, and (v) persistent infection [5,158,159,160].

Furthermore, it has been suggested that EV infection may also play a role in the development of thyroid diseases. Thyroiditis has been associated with an increased IgM and IgG against CVB [161,162]. In a previous study, EV RNA was detected in 27.3% and 24.3% of postoperative thyroid tissues obtained from patients with thyroiditis and patients with thyroid tumors, respectively; however, no association was found between the presence of EV infection and thyroiditis, lymphocytic infiltration, or the presence of circulating TPOAb in this study [163].

The EV genome has been found to be the most detected genome (51%) of the common viruses found in the thyroid glands of patients with GD or HT [164]. In addition, EV RNA or the capsid protein VP1 has been found more frequently in the thyroid tissue of patients with GD than in that of controls [165,166], and it has been found to be colocalized with protein kinase R within thyroid cells, suggesting that an antiviral tissue response may be a possible trigger for AITD [166]. EV strains isolated from AITD or T1D cases can suppress IFN pathways and the transcription of several cytokines in vitro while increasing, e.g., the transcription of IL18, JAK1/STAT1, known to play a pathogenic role in organ-specific autoimmunity [167,168]. It has been shown that CVB4 can replicate and persist in a human thyroid carcinoma cell line for up to 24 days post-infection and that it can induce the apoptosis of these cells [169], which is a process involved in triggering autoimmunity [170].

Maternal EV infection during pregnancy or maternal exposure to TPOAb in utero has been linked to the development of thyroiditis or AITD in offspring, and hypothyroidism has been shown to be significantly more frequent (60%) in children with IgM antibodies against EV than in controls [171,172].

## 8. Conclusions

Evidence of the presence of several other viruses in the endocrine organs is available for human T-cell lymphotropic virus-1, herpes simplex virus, rubella, mumps virus, Epstein–Barr virus, varicella zoster virus, cytomegalovirus, human foamy virus, and Simian virus 40, and these viruses have been associated with various endocrine diseases [173,174,175,176]. Viruses can transiently or permanently influence the functions of endocrine organs through direct damage of endocrine cells or through indirect mechanisms, especially through the activation of the host antiviral immune response, which may lead to the development of local or systemic inflammation or organ-specific autoimmunity that results in certain endocrinopathies. Since viral infections can cause endocrine disorders (Table 1), it would be interesting to determine whether the treatment and elimination of the infection could restore the host’s endocrine functions. Unfortunately, there are few clinical data in this regard. The recent direct-acting antivirals used in chronic HCV infection treatment have been shown to reduce not only liver-related mortality but also morbidity due to extra-hepatic manifestations, including several endocrine disorders [121,122,177]. A decrease in HIV-associated endocrinopathies since the widespread use of antiretroviral therapies has been reported [2,3]. However, despite the decrease in viral load, chronic inflammation persists and may be associated with many endocrine disorders, such as insulin resistance and diabetes mellitus [85,178,179]. More clinical data are needed to determine how to manage post-viral endocrinopathies in addition to symptomatic treatments. Further studies are also needed to clarify the pathophysiological mechanisms by which viral infections induce endocrine disorders in order to develop new strategies for their prevention and/or their treatment.

## Figures and Tables

**Table 1:** Impacts of viruses on endocrine organs.

Viruses	Affected Organs or Tissues	Diseases or Clinical Manifestations	Pathophysiological or Molecular Mechanisms	References
SARS-CoV	Hypothalamus, pituitary, and adrenal glands	HPA axis dysfunction	Alterations in adenohypophyseal endocrine cells	[14,15,16,17]
		Syndrome of inappropriate antidiuretic hormone secretion	High serum IL-6 levels Non-osmotic release of antidiuretic hormone	[18,19]
		Pituitary infarction	Coagulopathy, platelet dysfunction, and thrombocytopenia	[6,20]
		Adrenal insufficiency	Molecular mimicry between amino acid sequences of virus and host ACTH	[22]
	Thyroid	Thyrotoxicosis	High serum IL-6 levels	[23]
	Pancreas	Type 1 and type 2 diabetes Diabetic ketoacidosis	Structural, transcriptional, and functional alterations in infected insulin-producing pancreatic β cells	[9,44,46,49]
	Gonads	Hypogonadism	Virus-induced defective Leydig cell function Increased markers of inflammation	[55,56,57,58]
HIV	Pituitary gland	Alterations in growth hormone secretion Hypopituitarism Diabetes insipidus		[70,71,72,73,74]
	Adrenal glands Thyroid Gonads Pancreas	Adrenal insufficiency Hypothyroidism Hypogonadism Type 2 diabetes Insulin resistance, Lipodystrophy, Dyslipidemia	Opportunistic infections, weight loss, undernutrition, and direct cytokine effects on the gonads Systemic inflammation Alterations in adipokine levels and CD4+ and CD8+ T-cell function Increased microbial translocation	[3,74,75,76,78,79,80,85,86,87,88,89]
HCV	Thyroid Pancreas Gonads	Hypothyroidism Autoimmune thyroiditis Thyroid cancer Type 2 diabetes Insulin resistance Erectile dysfunction Alteration in spermatogenesis	Upregulation of IFN-γ and CXCL10 expressions Downregulation of cell surface expression of GLUT2 and impairment of glucose uptake in hepatocytes Excessive TNF-α response and high oxidative stress markers Increase in homocysteine and estrogen levels Low serum levels of inhibin B, insulin-like growth factor 1, and total testosterone	[94,95,96,97,98,101,102,103,104,105,106,107,111,112,115,116,117,118]
Orthohantaviruses	Pituitary gland Thyroid and gonads	Hypopituitarism Hormonal defects	Virus-induced necrotic and hemorrhagic damage Hypotension and/or vasospasms, thrombocytopenia, thrombopathy, and coagulation disorders Autoimmune mechanism	[128,129,130,131,132,133,134,135,136]
Parvovirus B19	Thyroid	Graves’ disease Hashimoto’s thyroiditis	PRDM1 upregulation, persistent infection, cell apoptosis, and increased inflammatory-related gene expression induced by the virus	[144,145,146,147,148,149]
Coxsackievirus B	Pancreas Thyroid	Type 1 diabetes Thyroiditis Autoimmune thyroid diseases	Molecular mimicry, bystander activation of pre-existing autoreactive T cells, alteration in central tolerance to β-cell antigens resulting from thymus infection, presence of non-neutralizing antibodies, and persistent infection Suppression of IFN pathways and transcription of several cytokines, increased transcriptions of IL18 and JAK1/STAT1, and apoptosis	[5,158,159,160,161,162,164,165,166,167,168,169,170]

## Data Availability

Not applicable.
